# In This Issue

**DOI:** 10.1002/cfg.316

**Published:** 2003-07


**Comparative and Functional Genomics**
*Comp Funct Genom* 2003; **4**: 365.
Published online in Wiley InterScience (www.interscience.wiley.com). **DOI:** 10.1002/cfg.316

### In this issue

### The yeast transcriptome on heat and cold shock

**Becerra *et al.*** exposed *S. cerevisiae* cells to a range of heat and cold shocks, including a pre-adaptation to heat shock, and then observed and contrasted the changes in gene expression in each case, identifying general and specific shock-related genes.

### Metabolic footprinting to differentiate *E. coli* mutants

**Kaderbhai** and **colleagues** used metabolic footprinting to successfully discriminate a set of *E. coli* tryptophan metabolism mutants, based on the metabolites they secrete into the medium.

### Special Section of selected reviews from the 4th Bologna Winter School on Bioinformatics: Hot Topics in Structural Genomics

**Rita Casadio** introduces the special section, with an overview of the topics discussed at the Winter School.

**Xin Yuan *et al.*** give us a background to *ab initio* protein structure prediction using folding pathway models and present their new pathway model, which uses a fragment library and a set of nucleation/propagation-based rules.

**Anna Tramontano** discusses recent advances in comparative (homology) modelling techniques, and the implications of the results from the CASP5 competition for tools and automated servers in this area.

**Pier Luigi Martelli *et al.*** describe their suite of programs designed to filter the proteomes of Gram-negative bacteria to find new membrane proteins.

**Patrick Aloy** and **Robert Russell** describe how data from complexes of known three-dimensional structure can be used to study and predict protein interactions and complexes.

**Fabrizio Ferrè** and **colleagues** discuss their development of computational tools for inferring protein interaction specificity rules, and functional annotation, using structural information.

**Rachel Bell** and **Nir Ben-Tal** discuss existing methods for the identification of biological inter-protein interfaces, and present their own tool, which uses phylogenetic trees built from multiple sequence alignments as input.

**Alfonso Valencia** discusses the use of multiple sequence alignments as tools for protein structure and function prediction, and in particular for predicting protein interactions.

**Susan Jones** and **Janet Thornton** review the current approaches for predicting DNA binding function in proteins and summarize their new method, published earlier this year.

**Naomi Siew** and **Daniel Fischer** discuss the issue of ORFans and poorly conserved ORFs; their origins, their abundance in various genomes, their potential for function, and what can be gained from their study.

**Silvia Saviozzi** and **Raffaele Calogero** discuss the comparative performance of software packages currently available for Affymetrix GeneChip microarray probe expression measures, data normalization and statistical validation.

### Conference calendar

A listing of genomics-related conferences planned for November 2003–January 2004.

Copyright © 2003 John Wiley & Sons, Ltd.

